# Atomistic insight into Al-Mg friction stir welding process via molecular dynamics simulation

**DOI:** 10.1371/journal.pone.0350194

**Published:** 2026-06-04

**Authors:** Van-Thuc Nguyen, Van Huong Hoang, Thanh Tan Nguyen, Tran Ngoc Thien, Xuan-Tien Vo, Van Thanh Tien Nguyen

**Affiliations:** 1 Faculty of Mechanical Engineering, HCMC University of Technology and Engineering, Ho Chi Minh City, Vietnam; 2 Faculty of Mechanical Engineering, Industrial University of Ho Chi Minh City, Ho Chi Minh City, Vietnam; Lovely Professional University, INDIA

## Abstract

Friction stir welding (FSW) has recently intrigued academics’ interest due to advances in high strength, low heat generation, tiny grain size, no melting, and the ability to produce dissimilar welding. It created a weld joint by stirring and applying stress to the interface of two metallic plates with a high-hardness tool. The impact of tool pin geometries during the stirring process and the existence of the friction stir welding tool are typically disregarded, particularly in molecular dynamics (MD) simulations, despite a large number of earlier reports. Therefore, in this report, the dissimilar welding joint of Mg-Al by friction stir welding (FSW) is investigated via MD simulation. The effects of travel speed, rotation speed of the tool, and tool pin geometry on the formation of Mg-Al weld joints are examined. The results indicate that Al atoms mix more effectively in the Mg plate than Mg atoms do in the Al plate. Improving the travel speed from 500 to 2000 m/s leads to a more severe atomic strain distribution, leading to a high-temperature zone up to 1000 K and an amorphous fraction of 18.8%. Moreover, the plastic deformation and high-temperature zone tend to become greater when the rotation speed increases. The mechanical atomic mixing level fluctuates with rotation speed, reaching a maximum value at 12 rad/ps. Rotating at 12 rad/ps also leads to the highest levels of mechanical atomic mixing and amorphous atoms. Considering the tool pin geometry, the rectangular prism and cone geometries gain a higher level of heating. The triangular and tube geometries create a better stirring effect. The results enhance atomic-level understanding of the mechanics of the FSW process. The following studies should evaluate the influence of offset distance and tool angle on the material flow of base metals.

## 1. Introduction

Recently, friction stir welding (FSW) has attracted many researchers due to the advances of high strength, low heat generation, fine grain size, no melting, and the ability to create dissimilar welding [1–3]. It created a weld joint by stirring and applying force with a high-hardness tool on the interface between two metallic plates. Besides, operating FSW also has many merits of energy saving, no filler, no shield gas, and low distortion [4,5]. Therefore, this technique is commonly applied in many industrial fields where light metal is preferred, such as automotive, aerospace, shipbuilding, and railways. Bharti *et al.* [6] examined the effect of FSW parameters on material microstructural changes and found that tool stirring, grain refining, and superplasticity improve joint mechanical properties and increase efficiency in the shipbuilding industry. To weld dissimilar metals such as Al-Cu, Al-Mg, and Al-steel, FSW is preferred as it can prevent the formation of brittle intermetallic phases during the melting process, leading to the high quality of the melt joint [7,8]. The reason is the reduction of the molten phase during the FSW process, which has the advantage of solid-state welding. Therefore, the formation of intermetallic phases, as happens in other fusion welding methods, is limited.

Mg is a light metallic material with low strength; joining with other materials, such as aluminum, can create a reliable structure. Fusion welding could lead to the formation of intermetallic phases, such as Mg₁₇Al₁₂ and Al₃Mg₂, dramatically reducing the strength of the structure. Many studies have considered the FSW process of Al-Mg dissimilar welding, as the intermetallic phase formation is prevented [9,10]. Morishige et al. [11] compared the weld joints created from laser welding and FSW processes. Due to the presence of the Mg₁₇Al₁₂ intermetallic phase in the weld bead, the laser welding joint is brittle. In reverse, the FSW joint between Mg and Al is strong enough for structural applications despite some intermetallic phases. The hardness of the FSW joint is much lower than that of the laser welding one, improving the welding efficiency to 61%. Overall, welding Mg-Al with FSW creates a much better welding quality compared to the laser welding method. Firouzdor *et al.* [12] applied FSW for 6061 Al and AZ31 Mg dissimilar welding, examining the effects of welding tool, travel speed, and rotation speed. The results showed that these parameters impact the heat input. Increasing the rotation speed and decreasing the travel speed gives rise to the heat input amount. Therefore, they impact the formation of intermetallic compounds and material flow. The high heat input could increase the material flow; however, the intermetallic formation is also improved. The optimal parameters reach 80‒100% welding efficiency.

Zettler *et al.* [13] also investigated the dissimilar welding between 6040 Al and AZ31 Mg via FSW. The welding efficiency is 80% compared to the base material. In the stir zone, there is complex plastic flow vortex and recrystallization. These phenomenon leads to the formation of fine grains and a small amount of intermetallic phases, explaining the relatively high strength of the weld joint. Firouzdor *et al.* [14] surveyed the impacts of tool position on the characteristics of FSW between Al and Mg. This study indicated that positioning the tool can greatly impact heat input and joint strength. Moreover, modifying lap joint welding can increase joint strength by two times. Fu *et al.* [15] surveyed the FSW of Al and Mg by considering the influences of the intermetallic compound. Increasing the heat input leads to a higher welding temperature. Thereafter, the intermetallic compound, including Al_3_Mg_2_ and Mg_17_Al_12_, becomes thicker due to the stronger level of solid-state mechanical atomic mixing. These brittle phases negatively influence the welding quality.

Zettler *et al.* [13] also investigated the dissimilar welding between 6040 Al and AZ31 Mg via FSW. The welding efficiency is 80% compared to the base material. In the stir zone, there is a complex plastic flow vortex and recrystallization. These phenomena lead to the formation of fine grains and a small amount of intermetallic phases, explaining the relatively high strength of the weld joint. Firouzdor *et al.* [14] surveyed the impacts of tool position on the characteristics of FSW between Al and Mg. This study indicated that positioning the tool can greatly impact heat input and joint strength. Moreover, modifying lap joint welding can increase joint strength by two times. Fu *et al.* [15] surveyed the FSW of Al and Mg by considering the influences of the intermetallic compound. Increasing the heat input leads to a higher welding temperature. Thereafter, the intermetallic compound, including Al₃Mg₂ and Mg₁₇Al₁₂, becomes thicker due to the stronger level of solid-state mechanical atomic mixing. These brittle phases negatively influence the welding quality.

The experimental studies are crucial in developing the FSW technique, but are limited by the cost and indirect observation. Besides, simulation might provide more insight into the FSW process with lower cost and micro to nano scale observation [16,17]. During the FSW process, the mechanical atomic mixing mechanism, plastic flow vortex movement, and interface evolution can be analyzed effectively. This is the reason why many authors investigated FSW via simulation. Mypati *et al.* [18], for example, examined the dissimilar welding of Al-Cu via molecular dynamics (MD) simulation. The merits of nanoscale observation via MD simulation provide more insight into the FSW process. The mechanical atomic mixing level showed that the Al atomic mixing level is higher than the Cu one. Moreover, besides the intermetallic compound and the original FCC structures, the amorphous phase also exists in the weld joint. Li *et al.* [19] also studied the FSW of Al-Cu dissimilar welding through MD simulation. The results showed that the mechanical atomic mixing increases when increasing the operation temperature. Remarkably, Medina *et al.* [20] applied MD simulation to survey the FSW between two Al plates. The study pointed out the advances of using MD simulation to mimic the FSW process, especially in material flow and temperature distribution. The twin boundary and high strain rate present in the weld joint significantly impact the welding quality. The operation procedure also has a strong impact on the formation of the FSW joints. Liu *et al.* [21] developed a new FSW process called vortex-FSW via computational fluid dynamics (CFD). The primary source of heat for welding is the material’s plastic deformation process. On various horizontal planes of the plastic flow vortex inside the workpiece, the volumetric heat generation rate first rises and then falls radially. The heat from FSW is mostly generated from the tool rotational speed, leading to the plastic deformation of the workpiece. In addition, the penetration depth can be calculated from the plastic flow vortex distribution on the bottom surface, preventing the risk of an incomplete penetration defect. Al-Mg alloying was examined by Kundu et al. [22] with respect to alloying temperature, atomic migration, coalescence kinetics, and mechanical properties at different heating rates. Heating rates and coalescence kinetics were presented to be significantly correlated, however mechanical properties were not greatly influenced. Goel et al. [23] studied the differences in friction stir spot welding between Al-Al and Al-Mg joints. The correlations found here between interfacial stress asymmetry, defect clustering, and local heating can be used to guide parameter selection and tool design for broader FSSW applications beyond the particular Al-Mg configuration.

Besides traditional metallic materials, FSW can also be applied to polymers and composites. Kumari *et al.* [24] concentrated on the fine-tuning and analysis of variance (ANOVA) analysis to indicate the critical factor during the FSW process. This study also noted that in the end stage, there is a hole in the weld, leading to the demand for further research. Besides ANOVA, Jha *et al.* [25] implied a hybrid approach method to optimize the surface quality during a metallic process. This model provides better surface roughness than the other ensemble learning models. Bandhu *et al.* [26], on the other hand, applied both Taguchi analysis and artificial neural networks (ANN) to increase the welding quality of A387 steel pipe. They conducted 25 experiments to increase the depth of penetration (DOP) while reducing the heat-affected zone and bead width values. The most influential parameter is welding voltage due to its direct relationship to the heat input. The results increase the quality of the welding process by introducing a multi-objective optimization. Fuse *et al.* [27] predicted the strength of the aluminum FSW weld. They used the machine learning (ML) method with a dataset of more than 200 samples, applying ML algorithms. The ML framework achieved a highest accuracy of 81.6% with the ABC model, indicating the advantages of ML methods in FSW. Nagendra *et al.* [28] trained a dataset of 360 cross-sections for predicting dissimilar laser welding strength via the ANN method. They applied the Orange data mining method to process the dataset, achieving both validity and predictive accuracy. A Multilayer Perceptron (MLP) neural network outperformed the others, achieving 94.9% accuracy. The findings demonstrate ANN as a reliable and scalable method for defect classification in steel-copper welding.

Despite numerous prior reports, the presence of the friction stir welding tool and the effect of tool pin, i.e.,s during the stirring process are usually ignored, especially in MD simulations. Prior reports mostly consider the friction between two base metals rather than the deformation of the base metal under the shoulder tool pressure. In this study, the effects of tool pin geometry, tool rotation speed, and travel speed are examined when simulating Mg-Al dissimilar welding. The weld joint formation evolution, temperature distribution, mechanical atomic mixing, structure transformation, plastic flow vortex, and surface morphology are analysed. The results could provide more atomistic insight into the mechanism of the FSW process.

## 2. Computational method

**Fig 1:**
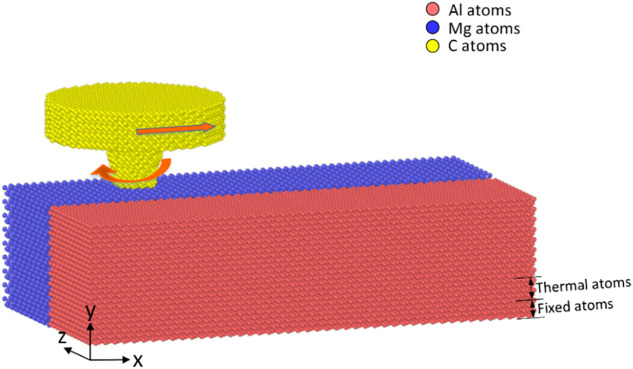
Al-Mg dissimilar FSW model..

The FSW model includes Al and Mg blocks, having the size of 190 Å *x* 50 Å *x* 50 Å, as shown in Fig 1. There is a small distance of 1 Å between the two blocks, representing the gap before welding. Periodic boundary conditions were applied in all directions to focus on local atomic-scale stirring behavior under severe shear stress. The outer shell, including the bottom layer and four side layers, with a thickness of 4 Å, is fixed to ensure the stability of the model. The next layer to the bottom layer with 4 Å thickness is the thermostat layer, while the upper layer for the FSW process is the Newtonian layer. The welding tool is created from diamond and treated as a rigid body, because its extremely high hardness compared to the Al and Mg blocks. The study surveys the impacts of travel speed, rotational speed, and tool pin geometry, as shown in Table 1. The travel speed is set at 500–2000 m/s, and the angular speed is set at 4–16 rad/ps to compensate for the system’s small dimension [20]. Moreover, this selection also helps ensure reasonable computation time for the study [29]. The extremely small time step of the MD simulation also requires a very fast speed to observe reasonable deformation during the friction stir welding process. In addition, the MD simulation is an atomic domain, which is different from the bulk samples. Therefore, to achieve the same total strain with nanoscale, this high speed is necessary. NVE ensemble was applied to ensure undisturbed atomic dynamics during the welding process and avoid artificial thermostat interference. While NVT is used for equilibration and boundary heat dissipation.

**Table 1:** Parameters of MD simulation in Al-Mg dissimilar FSW process.

Parameter	Value
Travel speed (m/s)	50, 100, 150, 200
Angular speed (rad/ps)	4, 8, 12, 16
Tool pin geometry	rectangular prism, cone, triangular prism, tube
Substrate temperature (K)	300
Timestep (ps)	0.001
Ensemble	NVE, NVT

The atomic interactions between Al and Mg atoms are defined by the EAM potential [30], while the interaction between Al-C and Mg-C is simulated by the LJ potential [31,32]. The LAMMPS program is applied to simulate the FSW model [33]. OVITO software is used to analyze the simulation outcomes and generate figures [34].

## 3. Results and discussion

### 3.1. Effects of travel speed

In this section, the tool moves at a travel speed range of 500–2000 m/s. Initially, it rotates and moves down toward the Al and Mg plates. Then it moves along the gap at different travel speeds and finally moves up to remove from the plates. The angular speed is set at 4–16 rad/ps to keep the same ratio between the travel speed and the rotational speed at all cases. Besides, the temperature is selected at 300 K, and the surface crystalline orientation is set at (001) vs (001).

**Fig 2:**
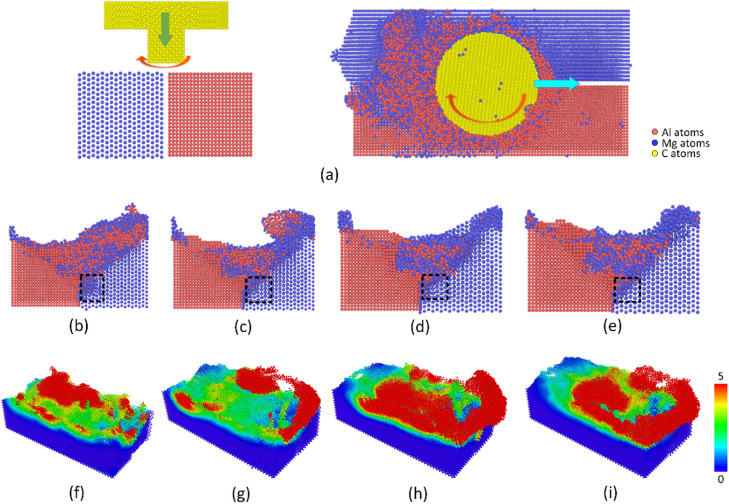
Surface morphology and shear strain distributions of the Al-Mg weld joint at various travel speeds: (a) welding process, (b) surface morphology – 500 m/s, (c) surface morphology – 1000 m/s, (d) surface morphology – 1500 m/s, (e) surface morphology – 2000 m/s; (f) strain distribution – 500 m/s, (g) strain distribution – 1000 m/s, (h) strain distribution – 1500 m/s, and (i) strain distribution – 2000 m/s.

Fig 2 displays the surface morphology and shear strain distribution of the Al-Mg at various travel speeds. After the FSW, the weld joint is generated, and the gap between the two plates disappears. The plastic deformation created by the tool and local melting phenomenon cause the mixing phenomenon between Al and Mg atoms, or a mechanical atomic mixing mechanism. Remarkably, the Mg atoms are more severely deformed than the Al plate, as shown in Fig 2(b)-(e), due to its lower hardness. Increasing the travel speed leads to a higher deformation rate. The shear strain distribution results also indicate that improving the travel speed from 500 to 2000 m/s leads to more severe atomic strain distribution, or a higher deformation rate, as shown in Fig 2(f)-(i). Interestingly, at the end of the weld line, there are vortices created by the rotation of the tool when it is removed from the plates. The mechanical atomic mixing level is discussed in the following results.

**Fig 3:**
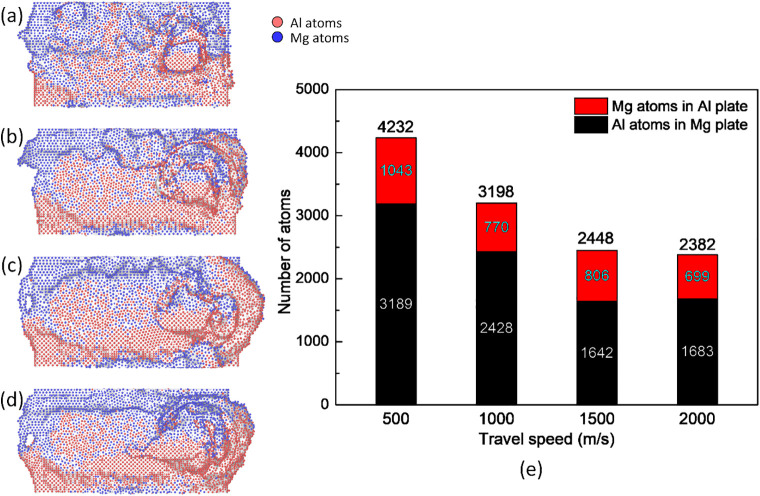
Weld joint surface and number of mechanical atomic mixing in the opposite plates of the Al-Mg weld joint at various travel speeds: (a) 500 m/s, (b) 1000 m/s, (c) 1500 m/s, (d) 2000 m/s, and (e) number of mechanical atomic mixing.

Fig 3 presents the weld joint surface and number of mechanical atomic mixing of atoms in the opposite plates in Al-Mg FSW. The number of mechanical atomic mixing is calculated in OVITO by counting the number of atoms of each type moving toward the other plate. After FSW, the Mg atoms on the Al side count the number of Mg atoms on the Al side, while the Al atoms on the Mg side count the number of Al atoms on the Mg side. The weld joint shows that increasing the travel speed mostly leads to a flatter weld line. Moreover, at the end of the weld line, there is a higher deformation level when increasing the travel speed due to the greater collision and torsion energy. Besides, the total number of number of mechanical atomic mixing atoms gradually decreases from 4232 to 3198, 2448, and 2382, corresponding to the travel speeds of 500, 1000, 1500, and 2000 m/s, as shown in Fig 3(e). The results show that increasing the travel speed mostly leads to a reduction in the number of Al and Mg atoms mixing in other plates. The reason is the lower processing time when increasing the travel speed, leading to a lower number of mechanical atomic mixing level. Therefore, the possibility of thinner intermetallic phase formation is lower. Interestingly, the thinner the interface, the lower the intermetallic thickness. Therefore, the welding quality increases when this brittle phase is not present in the structure, which is indicated in Venkateswaran and Reynolds’ report [35]. Hao *et al.* [36] also indicated that in their FSW study, increasing the travel speed also leads to better welding quality. Besides, the temperature distribution also impacts the possibility of the formation of the intermetallic phase. Atomic transport in FSW is driven by tool-induced rotation and severe shear deformation rather than thermally activated diffusion. Therefore, no diffusion coefficient was estimated in this work.

**Fig 4:**
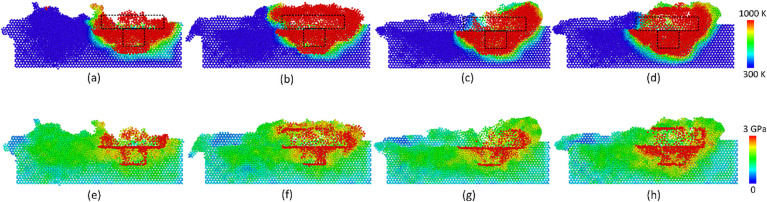
Temperature and stress distributions of the Al-Mg weld joint at various travel speeds: (a) temperature of 500 m/s, (a) temperature – 500 m/s, (b) temperature – 1000 m/s, (c) temperature – 1500 m/s, (d) temperature – 2000 m/s, (e) stress – 500 m/s, (f) stress – 1000 m/s, (g) stress – 1500 m/s, (h) stress – 2000 m/s. The color scale bar distributes colors to particles based on a given input parameter, providing a simple way to visualize local values using a rainbow color gradient. For temperature distribution, blue is related to 300 K, and red is related to 1000 **K.** For stress distribution, blue is related to 0, and red is related to 3 GPa.

Fig 4 displays the temperature and stress distributions of the Al-Mg weld joint at various travel speeds. The temperature results show that at the travel speed range of 500–2000 m/s, the temperature of the local zone around the tool could reach up to 1000 K. This temperature can lead to the local melting of the Mg plate, which facilitates the weld joint formation. The temperature differences between various travel speeds are not high. It is noteworthy that large differences in thermal conditions could reduce the quality of the weld joint, as it facilitates crack, slip, and intermetallic formation [37,38]. Moreover, Kim *et al.* [39] showed that the heat input is proportional to the rotation speed but in proportion to the travel speed. Liu *et al.* [21] also indicated that the plastic deformation caused by the tool movement is the main source of heat generation. In this section, the increase in travel speed does not lead to a sudden change in the temperature distribution due to the increase in the rotation speed at the same time. Besides, the stress distribution indicates a similar phenomenon, which shows that the high-stress zone concentrates around the tool. Interestingly, the atoms that stick to the tool surface reach an extremely high internal stress level due to the severe plastic deformation, as shown in Fig 4(e)-(h). The high-stress zone could perform as a potential position for failure modes like crack initiation. In other words, the residual stress can improve the possibility of the defect regions becoming failures.

**Fig 5:**
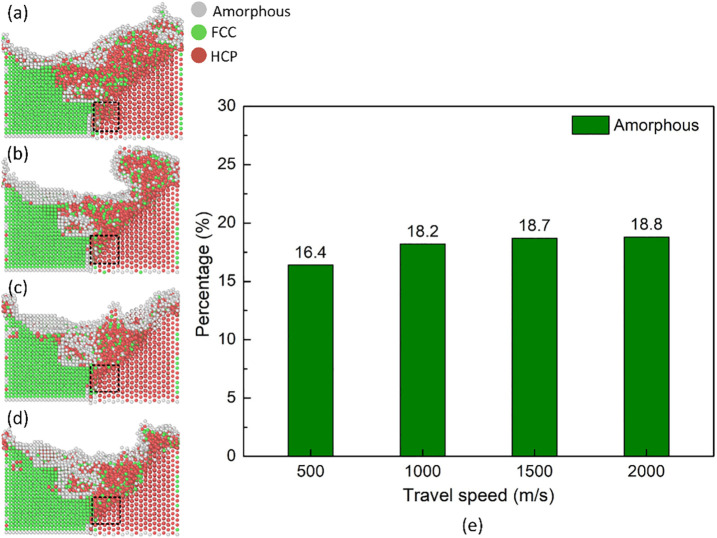
Structure transformation distribution and amorphous fraction of the Al-Mg weld joint at various travel speeds: (a) 500 m/s, (b) 1000 m/s, (c) 1500 m/s, (d) 2000 m/s, and (e) amorphous fraction.

The phase transformation in the FSW joint can identify the deformation level of the joint. They are local amorphous zones with very high strength and hardness, working as a dislocation barrier that can improve wear resistance and local strengthening. Therefore, it works as a transient deformation accommodation mechanism. These localized amorphizations present the intensity of plastic deformation in the stir zone and contribute to grain refinement rather than working as a detrimental phase. They can occur due to rapid strain rate and simulated cooling and have an indirect impact on the weld quality. Fig 5 displays the structure transformation distribution and amorphous fraction of the Al-Mg weld joint at various travel speeds. The phase transformation in the Mg plate penetrates deeper than the Al one, as shown in Fig 5(a)-(d), which is similar to the surface morphology result. The amorphous fractions are 16.4, 18.2, 18.7, and 18.8%, corresponding to the travel speed of 500, 100, 1500, and 2000 m/s. This result is similar to the shear strain result; increasing the travel speed leads to a higher ratio of amorphous phase. The reason is the higher plastic deformation level when increasing the travel speed of the tool. Moreover, the transformation zone penetrates deeper into the Mg plate rather than the Al plate, despite Al being the advancing side. This is because the Mg plate is softer and has a lower melting point compared to the Al plate. Generally, increasing the travel speed results in higher deformation and structure transformation levels. However, the number of mechanical atomic mixing level reduces when increasing the travel speed of the tool. Although intermetallic phases are known to be important in the FSW of Mg–Al, they were not directly tracked in this MD study. The formation of stable intermetallics is a diffusion-controlled process that happen over time scales, which is far beyond those accessible to MD. Instead, the simulations focus on early-stage atomic mixing and shear-induced interfacial disorder, which act as precursors to the intermetallic phases that can be observed experimentally.

### 3.2. Effects of rotation speed

In this section, the tool moves at a fixed travel speed of 1000 m/s, and the angular speed is set at 4‒16 rad/ps, changing the ratio between the travel and the rotation speed. Besides, the temperature is 300 K, and the surface crystalline orientation is set at (001) vs (001).

**Fig 6:**
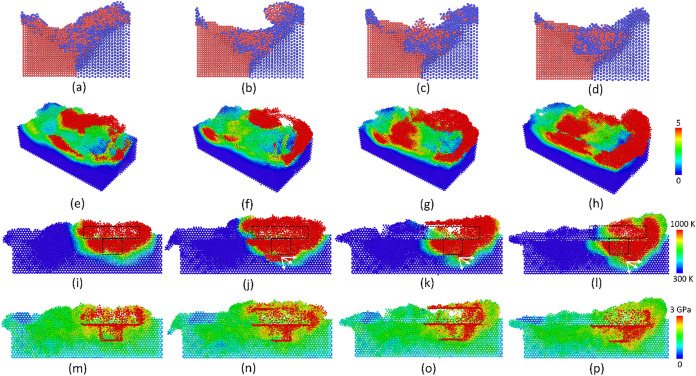
Surface morphology, shear strain, temperature, and stress distributions of the Al-Mg weld joint at various rotation speeds: (a), (e), (i), (m) 4 rad/ps, (b), (f), (i), (n) 8 rad/ps, (c), (g), (k), (o) 12 rad/ps, and (d), (h), (l), (p) 16 rad/ps.

Fig 6 displays the surface morphology, shear strain, temperature, and stress distributions of the Al-Mg weld joint at various rotation speeds. The weld joint was successfully formed after the FSW process. Moreover, increasing the rotation speed leads to a higher rate of shear strain level due to the higher plastic deformation rate. Similarly, when the rotation speed increases, the high-temperature spreads further due to the more dramatic plastic deformation level. Because the rotation movement creates most heat input [21], increasing the rotation speed gives rise to a higher temperature around the tool. This result is consistent with Kim *et al.* [39] study, indicating the higher heat input when increasing the rotation speed. The high-temperature zone can be high enough to melt the Mg plate, increasing the welding quality. Notably, Li *et al.* [19] indicated that the higher temperature also facilitates the diffusion process. The stress distribution indicates that the high-stress zone concentrates around the tool and along the weld line. It also exhibits in a narrow area, reducing the negative impact on the weld joint.

**Fig 7:**
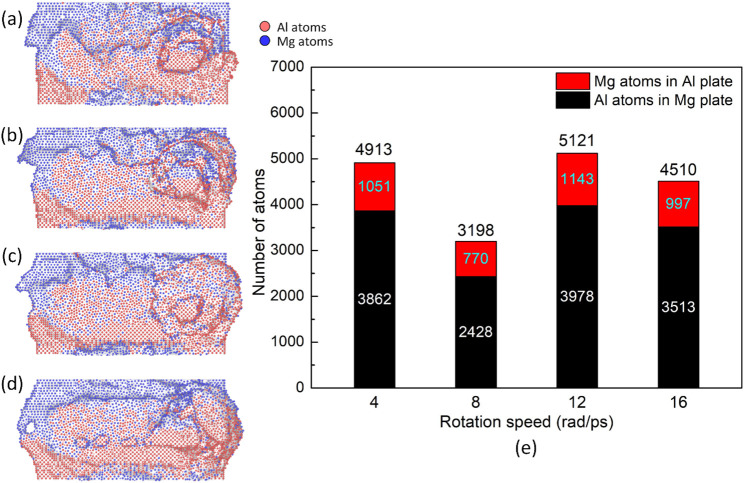
Weld joint surface and number of mechanical atomic mixing in the opposite plates of the Al-Mg weld joint at various rotation speeds: (a) 4 rad/ps, (b) 8 rad/ps, (c) 12 rad/ps, (d)16 rad/ps, and (e) number of mechanical atomic mixing.

Fig 7 presents the weld joint surface and number of mechanical atomic mixing of atoms in the opposite plates in Al-Mg FSW at various rotation speeds. Similar to the increase in travel speed, increasing the rotation speed mainly leads to a flatter weld line. At the end of the weld line, there is a twist deformation zone where the tool is removed after welding. The total number of mechanical atomic mixing is 4913–3198, 5121, and 4510, corresponding to the rotation speed of 4, 8, 12, and 16 rad/ps. The number of mechanical atomic mixing fluctuates when changing the rotation speed, reaching the highest value of 5121 atoms at 12 rad/ps. The local melting at the contact surface during FSW can be the reason for this fluctuation, as the thermal cycle is related to all factors, for instance, the heat input caused by travel and rotation movement, the friction coefficient variation, and the heat transformation of the base metals [40].

**Fig 8:**
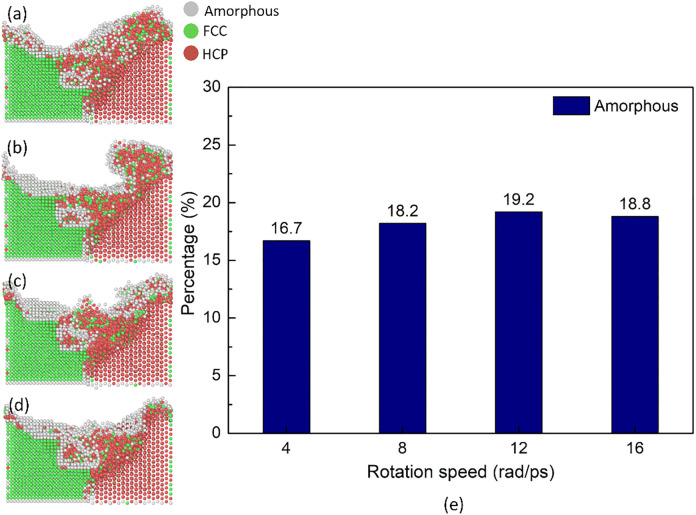
Structure transformation distribution and amorphous fraction of the Al-Mg weld joint at various rotation speeds: (a) 4 rad/ps, (b) 8 rad/ps, (c) 12 rad/ps, (d)16 rad/ps, and (e) amorphous fraction.

Fig 8 displays the structure transformation distribution and amorphous fraction of the Al-Mg weld joint at various rotation travel speeds. After FSW, many crystalline atoms are transformed into the amorphous phase. The presence of an amorphous phase is consistent with Mypati *et al.* [18] study, which points out that the amorphous zones develop locally during the FSSW because there is no chemical reaction between Al and Cu or crystallization. The amorphous fractions are 16.7, 18.2, 19.2, and 18.8%, corresponding to the rotation speed of 4, 8, 12, and 16 rad/ps. This result is similar to the number of mechanical atomic mixing result at 12 rad/ps, presenting the highest amorphous fraction of 19.2%. Despite the higher heat input level, dynamic recrystallization of the weld joint occurs due to the heat, and the cooling can recover the crystalline structure [41]. Therefore, the variation of the amorphous fraction is small when increasing the rotation speed. Overall, the high-temperature spreads further due to the more dramatic plastic deformation level when the rotation speed increases. Rotating at 12 rad/ps leads to the highest levels of number of mechanical atomic mixing and amorphous atoms.

### 3.3. Effects of tool pin geometry

The tool pin geometry strongly impacts the FSW. An optimal tool can create good stir deformation and increase the welding quality. In this section, the effect of tool pin geometry, including rectangular prism, cone, triangular prism, and tube, is investigated. Hassanifard *et al.* [42] showed that a cone tool pin geometry with an angle of 5‒20° possesses the optimal welding quality; therefore, the cone in this section is designed with an angle of 12.5°. The tool moves at a travel speed range of 1000 m/s, the angular speed is set at 8 rad/ps, the temperature is 300 K, and the surface crystalline orientation is set at (001) vs (001).

**Fig 9:**
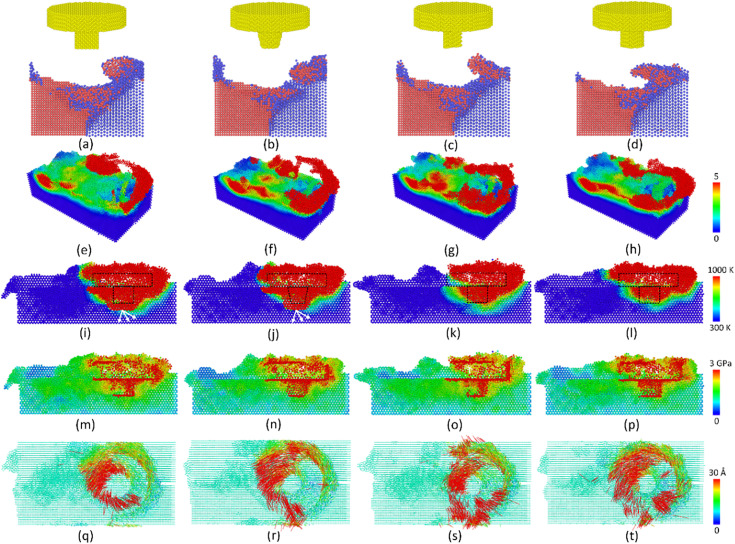
Surface morphology, shear strain, temperature, stress, and displacement vectors distributions of the Al-Mg weld joint with different tool pin geometrys: (a),(e),(i),(m),(q) rectangular prism, (b),(f),(i),(n),(r) cone, (c),(g),(k),(o),(s) triangular prism, and (d),(h),(l),(p),(t) tube.

Fig 9 displays the surface morphology, shear strain, temperature, stress, and displacement vectors distributions of the Al-Mg weld joint with different tool pin geometrys. With different tool pin geometrys, the stirring behavior could change significantly. All the tool pin geometrys, including rectangular prism, cone, triangular prism, and tube, can successfully generate the weld joints. Notably, the rectangular prism and cone tools have a larger high-temperature zone, indicating a higher heat input effect. The high-temperature zone spreads deeper toward the bottom of the pin in the rectangular prism and cone tools, as shown in Fig 9(i)-(j). Along the weld line and around the tool, the high-strain, temperature, and stress zones appear due to the drastic deformation caused by the stirring process.

In addition, the displacement vector result shows that the cone, triangular prism, and tube create a stronger plastic flow vortex than the rectangular prism tool pin geometry, as shown in Fig 9(q)-(t). In MD simulations of FSW, a plastic flow vortex is an atomic flow that presents solid-state plastic flow and material mixing under the severe shear deformation generated by the rotation of the tool, rather than molten material flow. Medina *et al.* [20] also showed that the tool characteristics significantly impact the materials flow during the FSW process. The following results will give more insight into number of mechanical atomic mixing and structure transformation.

**Fig 10:**
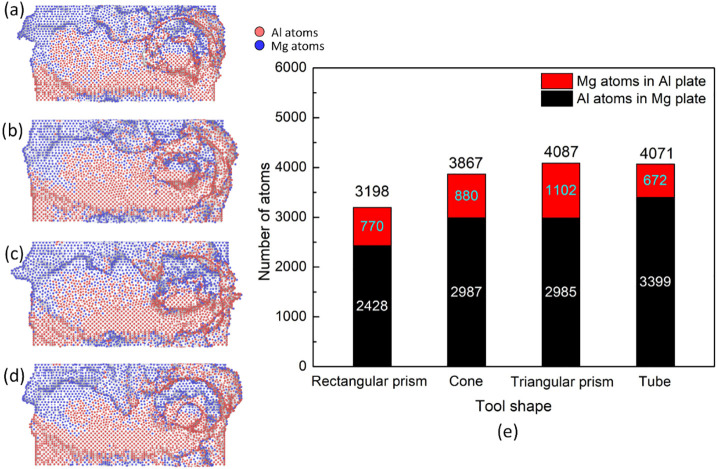
Weld joint surface and number of mechanical atomic mixing in the opposite plates of the Al-Mg weld joint with different tool pin geometrys: (a) rectangular prism, (b) cone, (c) triangular prism, (d) tube, and (e) number of mechanical atomic mixing.

Fig 10 presents the weld joint surface and number of mechanical atomic mixing of atoms in the opposite plates in Al-Mg FSW at various rotation speeds. Similar to the increase in travel speed, increasing the rotation speed mainly leads to a flatter weld line. At the end of the weld line, there is a twist deformation zone where the tool is removed after welding. The total number of mechanical atomic mixing is 3198, 3867, 4087, and 4071, corresponding to the tool pin geometrys of rectangular prism, cone, triangular prism, and tube. The mechanical atomic mixing numbers created by the triangular prism, cone, and tube are higher than the rectangular one, indicating a better stirring phenomenon. Especially, the highest mechanical atomic mixing are achieved in the case of a triangular prism due to the better blending and higher friction intensity. Vinith *et al.* [43] proved that a tool pin with an enhanced stirring effect could increase the welding quality.

**Fig 11:**
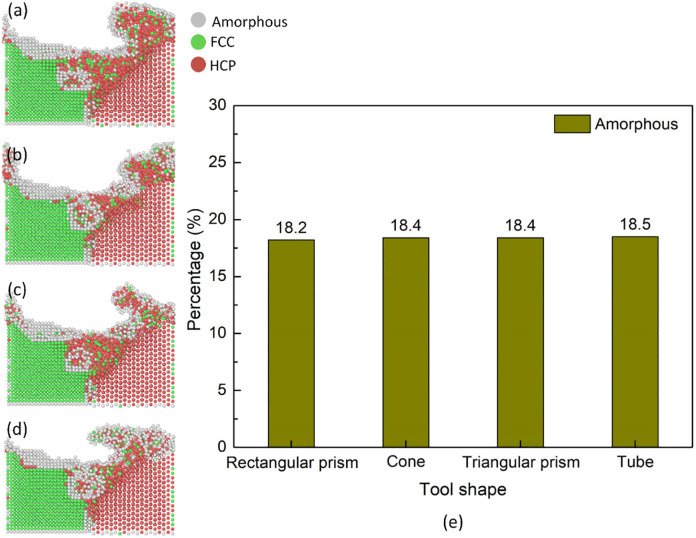
Structure transformation distribution and amorphous fraction of the Al-Mg weld joint with different tool pin geometrys: (a) rectangular prism, (b) cone, (c) triangular prism, (d) tube, and (e) amorphous fraction.

Fig 11 displays the structure transformation distribution and amorphous fraction of the Al-Mg weld joint with different tool pin geometrys. After FSW, many crystalline atoms are transformed into the amorphous phase. The amorphous fractions are 18.2, 18.4, 18.4, and 18.5%, corresponding to the tool pin geometrys of a rectangular prism, cone, triangular prism, and tube. This result is close to the number of mechanical atomic mixing results, as shown in Fig 10, indicating that the rectangular prism shape creates a lower number of mechanical atomic mixing level than the other shapes. In general, rectangular prism and cone tools have better heating effects but lower number of mechanical atomic mixing level and amorphous fractions. In reverse, triangular and tube tools have good stirring effects.

Despite the absolute process parameters being elevated due to MD time-scale constraints, the dominant behavior is still controlled by comparable dimensionless measures such as shear strain and strain rate. Therefore, the analysis in this study focuses on relative trends, which remain consistent and robust across the investigated parameter range. According to the results, the observed trends and mechanisms‒such as the dynamic recrystallization mechanism, the enhanced stirring effect of the tool pin, the rapid increases in heat input as rotation speed increases, the low value of the heat-affected zone, and the increase in travel speed‒could result in better mixing quality. Therefore, the validation in this study is conducted at the mechanistic and trend level. This qualitative agreement supports the physical reliability of the MD results.

## 4. Conclusion

This study investigates the impacts of tool pin geometry, tool rotation speed, and travel speed on Mg-Al dissimilar welding simulations. The weld joint formation evolution, number of mechanical atomic mixing, structural modification, and surface morphology are investigated. The results show that the Al atoms mix in the Mg plate more strongly than the Mg atoms mix in the Al plate. However, the Mg atoms mix deeper in the weld joint than the Al atoms. Improving travel speed from 500 to 2000 m/s causes a more severe atomic strain distribution, resulting in a high-temperature zone of up to 1000 K and an amorphous fraction of 18.8%. Furthermore, as the rotation speed increases, the plastic deformation and high-temperature zone expand. Atomic number of mechanical atomic mixing varies with rotational speed, peaking at 12 rad/ps. Rotating at 12 rad/ps produces the highest amounts of mechanical atomic mixing and amorphous atoms. In addition, rectangular prism and cone tools heat up more than triangular and tube shapes. The study results reveal atomic mechanisms of dynamic recrystallization, probing possible defect dynamics under massive strains, and studying interface formation and mixing at the atomic level. The results also identify the early stages of grain refining and inform mesoscale models with strong constitutive data. Despite several advantages, this study’s limitations include the model’s small scale and the simplified tool pin geometry. In further studies, the impact of offset distance and title angle of the tool should be considered to clarify their impacts on the material flow of the base metals. Future work should also consider repeated simulations to further assess statistical variability.

## Supporting information

S1 File The influences of the travel speed of tool pin on the deformation, local strain, stress, and temperature of Al-Mg FSW. (DOCX)

S2 File The influences of angular speed of the tool pin on the deformation, local strain, stress, and temperature of Al-Mg FSW. (DOCX)

S3 File The influences of tool pin shape on the deformation, local strain, stress, and temperature of Al-Mg FSW. (DOCX)

S4 File Supporting Information_Raw Data set. (ZIP)
