# Supplementary material for: Atomistic insight into Al-Mg friction stir welding process via molecular dynamics simulation
Source: PLoS One. 2026 Jun 4;21(6):e0350194. doi: 10.1371/journal.pone.0350194 (PMC13235913; doi:10.1371/journal.pone.0350194)
Supplement: S2 File — (DOCX) [file pone.0350194.s002.docx]

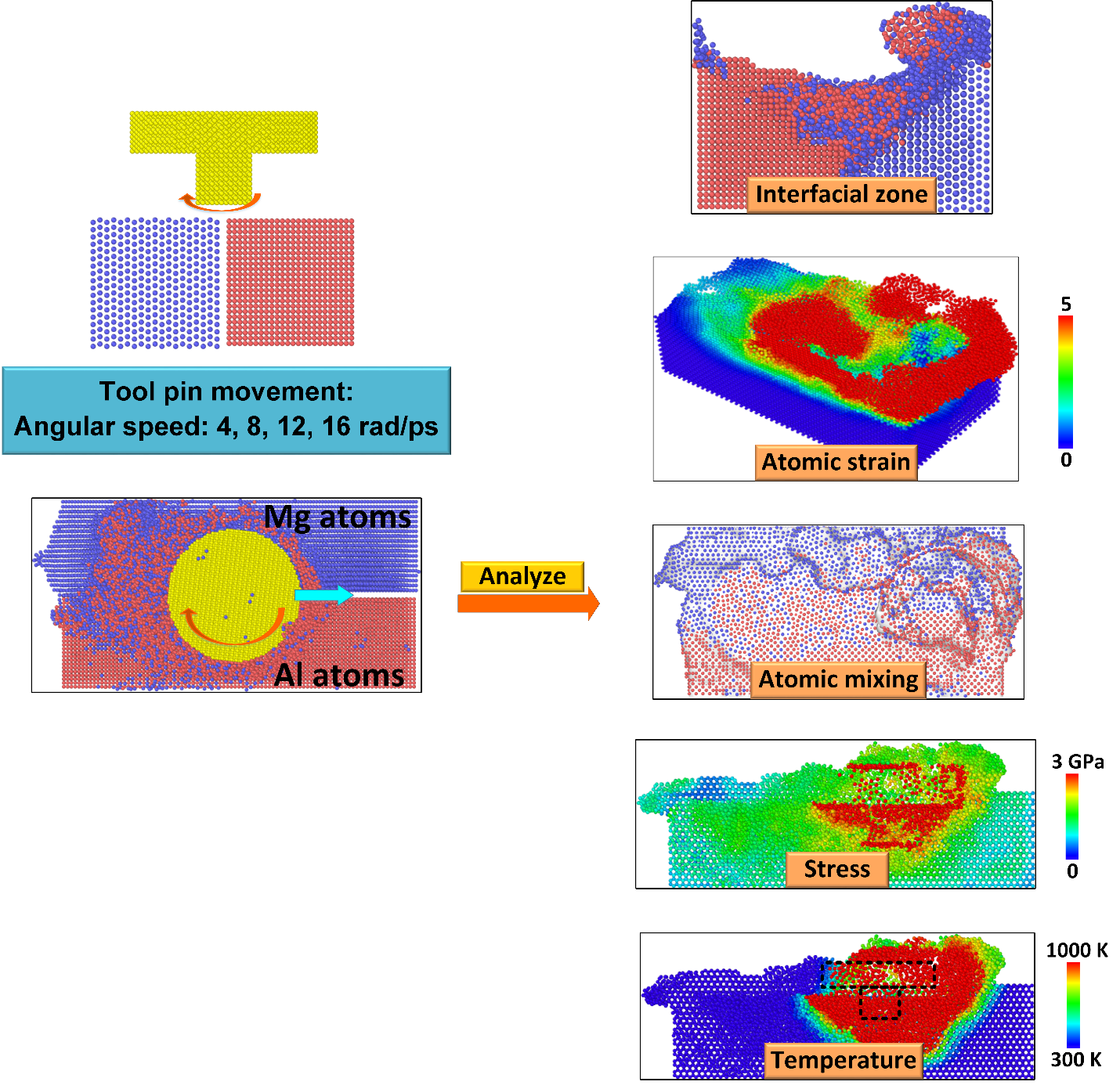


**S02.** The influences of the angular speed of the tool pin on the deformation, local strain, stress, and temperature of Al-Mg FSW.
