# Supplementary material for: Atomistic insight into Al-Mg friction stir welding process via molecular dynamics simulation
Source: PLoS One. 2026 Jun 4;21(6):e0350194. doi: 10.1371/journal.pone.0350194 (PMC13235913; doi:10.1371/journal.pone.0350194)
Supplement: S4 File — (ZIP) [file pone.0350194.s004.zip › Supporting Information_Raw Data set/Cover letter - raw Data.docx]

Dear Editors and Readers:

Thank you for your kind request.

We thank the reviewer for this helpful suggestion. In this study, the simulation .dump files generated by LAMMPS were all post-processed using OVITO to extract atomic-scale structural and visualization data. Therefore, to support the reproducibility of the figures, we provide representative .dump files for three main sections of the manuscript and the procedure used to construct the graphs. These materials are provided as supplementary data to allow verification of the analysis process. The values reported in the manuscript are therefore derived from post-processing procedures rather than being direct raw outputs stored separately from the simulation files. As these quantities are extracted from the .dump files through OVITO-based analysis, no additional raw data files exist beyond the original dump outputs. It means that, as they are extracted from the raw .dump file via OVITO, we cannot provide any intermediate files.

The general guidance and some detailed calculations:

+ https://www.ovito.org/docs/current/reference/pipelines/modifiers/index.html

+ Atomic strain:https://www.ovito.org/docs/current/reference/pipelines/modifiers/atomic_strain.html#particles-modifiers-atomic-strain

+ Displacement vector:https://www.ovito.org/docs/current/reference/pipelines/modi…cement_vectors.html#particles-modifiers-displacement-vectors

+ Color coding, temperature, and stress distribution:https://www.ovito.org/docs/current/reference/pipelines/modifiers/color_coding.html#particles-modifiers-color-coding

Thank you for reading.
